# An Introspective Approach: A Lifetime of Parkinson’s Disease Research and Not Much to Show for It Yet?

**DOI:** 10.3390/cells10030513

**Published:** 2021-02-28

**Authors:** Gordon W. Arbuthnott

**Affiliations:** Brain Mechanisms for Behaviour Unit, Okinawa Institute of Science and Technology, Graduate University, Onna-son, Okinawa 904-0495, Japan; gordon@oist.jp

**Keywords:** Parkinson’s disease, alpha-synuclein, genetics, deep brain stimulation, organoids, anatomy

## Abstract

I feel part of a massive effort to understand what is wrong with motor systems in the brain relating to Parkinson’s disease. Today, the symptoms of the disease can be modified slightly, but dopamine neurons still die; the disease progression continues inexorably. Maybe the next research phase will bring the power of modern genetics to bear on halting, or better, preventing cell death. The arrival of accessible human neuron assemblies in organoids perhaps will provide a better access to the processes underlying neuronal demise.

## 1. The Start of Something New

My interest in neuroscience started in the company of inquisitive psychiatrists wondering about the origins of schizophrenia. The ideas frequently discussed with my fellow worker and teammate Tim Crow were about the role of dopamine in the type I symptoms of schizophrenia, which are characterized by delusions, hallucinations, and erratic and disorganized speech and thinking. We were confident that dopamine had something to do with the disease; the problem was that the dopamine receptor blockers used to aid schizophrenic symptoms could result in a condition similar to Parkinson’s disease (PD) [1,2].

### 1.1. A Model and a Misleading Feedback 

Intrigued about dopamine, I departed, thanks to the Welcome Trust, for a postdoctoral year in Stockholm to participate in the development of the first animal model of PD. That happened more than a decade before an addictive drug contaminant (MPTP) turned out to be a potent neurotoxin of dopamine cells and caused Parkinsonism [3]. In Sweden, we observed that a local injection of 6-hydroxy dopamine (6-OHDA), unilaterally into the substantia nigra of adult rats, destroyed dopamine cells and not much else [4]. Undisturbed, rats that had received unilateral 6-OHDA could not be distinguished from sham animals, but if perturbed by handling or pinching their tails or by injecting them with amphetamine, the animals circled to the side ipsilateral to the lesion [1]. At the time, it was thought that brain dopamine levels were controlled by a feedback loop running from striatum to the dopamine cells and back. The “Amine Group” I worked with then interpreted their results on dopamine turnover with that in mind [5]. Back in Scotland, at the M.R.C. Brain Metabolism Unit, my students and I traced the “feedback loop” to determine its beginning and ending sites. However, in spite of using the long and difficult autoradiographic method, we could not convince ourselves that the return pathway ended on the dopamine-containing cells in substantia nigra pars compacta [6]. Still uncertain, we damaged striatal axons travelling from striatum to substantia nigra in the crus cerebri and, although the animals had clear behavioral symptoms that agreed with the work in Stockholm, the turnover of dopamine was modified to a normal extent by the dopamine antagonist, haloperidol [7]. The pathway from striatum was the output, not a feedback loop as suggested by earlier work showing that striatal output was responsible for the turning behavior [8] and later also supported by the group of DiChiara in Italy [9], who showed that destruction of striatal neurons did not affect the “feedback”.

### 1.2. Consequences, Predictions and Observations

The paper of Garcia-Munoz, et al. [7] was one of the many consequences of trying to “put the dopamine neurons back in the brain” [10]. In those early days, we had none of the imaging tools we take for granted today. The dopamine cells and their terminals were visible, but not their travelling axons. Only catecholamine neurons were fluorescent, so both their postsynaptic targets and their input connections were invisible [11]. As the anatomical results came in [12,13,14], there were arguments about which of the output systems were responsible for the turning behavior that was so useful for testing drugs, but whose relevance to normal movement, or most importantly PD, was still deficient. In spite of a chorus of support for brain stem areas as the source of the turning [15,16], we were skeptical. We predicted, instead, that the striatal command for turning, converged in the ventral thalamic nuclei, the gathering site of outputs from substantia nigra reticulata. Indeed, specific thalamic lesions, reduced the typical turning behavior following 6-OHDA [17]. For us, it was also important to determine if the animal models of PD also suffered a variety of cognitive- and sensory-related symptoms. As expected, we observed a profound neglect contralateral to the lesioned side, associated with an inability to learn new motor tasks with the paw contralateral to the lesion [18]. This set of experiments about dopamine’s action in brain was the continuation of the earliest ideas that Tim Crow and I developed in Aberdeen [19] linking dopamine to self-stimulating behavior [20,21]. 

## 2. The Steppingstones

### 2.1. Dopamine and Synaptic Structure

Cali Ingham, an electron microscopist, joined Edinburgh just as she completed her PhD in Oxford. She was interested in the formation of new synapses in the adult brain as described by Raisman and Field [22]. Then, it was clear that the heads of spines on medium spiny neurons were contacted by cortex and thalamus and that a proportion of spines also received a second synaptic input proposed to be dopaminergic [23]. With this in mind, we wrote a grant that would allow us to study what replaced dopaminergic synapses on spines after damage to dopaminergic afferents. After setting up everything for the experiment, Cali said: “I suspect that when the dopamine is gone—so are the spines”. That was a difficult thing to conclude… we needed to count dendritic spines! Indeed, months later, we knew there were spines lost—and not resulting from the time it took us to count. Age-matched older controls had fewer spines than young animals, but the 6-OHDA lesioned side had fewer still. More than 1400 dendrites later, the result was published [24], and we went on to show that the loss of inputs was even more extreme in human post mortem brains from PD patients [25]. 

While we were involved in these details, one of the great advances in the dopamine field came. Two output pathways from striatum, with different dopamine receptors on each, were proposed [26,27]. The idea led to a revolution in the way the basal ganglia was conceived, reported on in textbooks and used in the clinic. Some basic scientists had serious doubts that the system was as simple as it seemed, but the literature about dopamine in the brain took a new path for sure [10].

Then, we began to count dendritic spines again, this time differentiating between the two pathways from striatum. Since that original paper had interested others, by the time we had a possible answer, the world had joined us and we published together. The article included results from four laboratories concluding that out of the D1- and D2-expressing striatal medium spiny neurons, the D2-medium spiny neurons lost spines first [28]. Today, it is clear that both output pathways lose connections from the cortex, but that it takes longer for D1-expressing neurons, the direct pathway, to lose their spines [29,30]. These results biased my thinking toward a view of dopamine loss, as a way to study how the brain compensates for the damaged system. It could be that at least some symptoms result from inappropriate reactions to the loss, rather than the primary actions of dopamine.

### 2.2. Dopamine and Synaptic Strength

When Jeff Wickens visited my laboratory in Edinburgh, he suggested we take a look at the actions of dopamine in striatum from a different point of view. His theory that dopamine would act specifically on recently active cortical inputs to the striatum resulted in a series of experiments involving intracellular recordings from rat brain slices. Cortical stimulation produced long-term depression (LTD), and application of dopamine along with the stimulation converted LTD to a long-term potentiation (LTP) [31]. This led us to conclude that when dopamine release coincides with the activation of synapses, neuronal ensembles may emerge associated to a particular reinforcing event—a result in agreement with Wolfram Schultz’s famous evidence that dopamine may carry the reinforcement error signal [32]. Perhaps, striatal dopamine selects particular recently active cortical inputs for strengthening, in order to generate assemblies of striatal output neurons to carry the signals of reinforced behavior [33].

### 2.3. The Functional Striatal Anatomy and Its Consequences for Patients

One of the most important consequences of the two output systems was the advent of deep brain stimulation (DBS) for PD. The model of PD proposed that the D2 cells of the output pathway were highly active, so they would inhibit globus pallidus which would in turn, disinhibit the subthalamic nucleus (STN). This increased activity of the excitatory STN was expected to result in the bradykinesia. Therefore, removing the STN should reduce the symptoms. Indeed, it worked in monkeys, although it turned out to be a very risky surgery in humans [34]. This surgical approach closely bordered the internal carotid artery, resulting in a high risk of the strokes well known to cause hemiballismus [35]. Indeed, for a few days after the initial surgeries in monkeys, ballism was observed [34].

Following the suggestion that overactivation of STN should silence the neurons, it was proposed that its stimulation in patients should improve their condition at least by alleviating the worst of the motor symptoms [36]. That worked indeed, and it still does relieve motor problems for patients. Dieter Jaeger and I had three reasons to doubt the explanation for the success: 1. STN firing rates can be very high, sometimes recorded nearly 3 times faster than the “blocking” stimulation rate; 2. the stimulation pulse widths were too short to be likely to stimulate cell bodies; and, 3. the 50 µsec pulses were more efficient at activating myelinated fibers of which there were many around the stimulation site. When we tested the idea that myelinated axons were the source of the improvement, indeed we observed that simulation in the region of the STN induced a clear antidromic excitation of the rat’s pyramidal cortical neurons, whose axons pass close to the stimulation site [37]. Subsequently, Dieter and I followed different strategies in our separate parts of the world. With new colleagues in New Zealand, I was able to show that rats recovered from dopamine-blocker-induced akinesia as a result of stimulation in the subthalamus. Successful recovery required an intensity close to the threshold for the antidromic evoked potentials in cortex [38]. Moreover, cortical recordings from intact and 6-OHDA-treated freely moving rats revealed that the STN stimulation threshold for induction of antidromic evoked potentials and recovered mobility in lesioned animals close to controls [39]. A finding we thought would be of interest to neurologists was that the stimulation that increased movement also reduced cortical synchronization at beta frequencies [40]. Dieter in Atlanta went on to show that the fibers of the hyperdirect pathway from cortex to STN could support similar recovery in mice when stimulated with optogenetic techniques [41]. This cortical involvement in the generation of symptoms is still an ongoing field of interest in ameliorating the symptoms of PD [42,43,44,45,46,47]. Moreover, it also led to a flurry of theoretical work [48,49,50,51,52] and to confirmation of the earlier reports that antidromic activity could be found in patients receiving DBS [43,53,54,55,56,57]. It also suggested that, in spite of less than exciting trials of cortical stimulation in humans, mimicking the stochastic antidromic stimulation with DBS might be therapeutically useful, at least in the rat model [58]. Nonetheless, since the rodent cortex has no folds and is much thinner than the human one, a direct transfer of technology may not be possible. 

Further, while we were building up the account of the basal ganglia loops, we found their final return to cortex from the thalamic area [59]. The axons arrived in layer one, but in 1990, the cortical layer 1 was a “tar pit”, with a physiology which was impossible to elucidate. After my move to Japan, more recent work on this surface layer of the cortex is beginning to suggest another reason to consider cortex as part of the underlying problems of PD [60,61,62]. Layer 1 of cortex has relatively few inhibitory neurons and very many axonal terminals from other areas of the brain, including the ventromedial thalamus and other cortical areas. The dynamics among the cells suggest complex arrangements of the inhibitory cells displaying widespread but differential responses to stimuli in awake mice [63]. Since the inhibitory neurons are electrically coupled [64], this arrangement is a likely source of synchronous waves of activity that may underlie the EEG. In PD, EEG changes have been recorded that include more power in the beta frequency range. The cortical antidromic activity we saw in animal models also disrupted the very same EEG changes while recovering the animals’ movements [37,40]. Furthermore, in experiments on automatic control of DBS, Bergman’s group [65] suggested that triggering on the EEG beta frequencies led to the best control of the patients’ movements. Recent work also suggests that the sleeplessness of PD might also be related to the increased beta frequencies [66].

Meanwhile, the idea of the two functionally separated striatal output systems was in various kinds of trouble. Firstly, Costa [67], Cui et al. [68], da Silva et al. [69], and Tecuapetla et al. [70] showed that both groups of striatal cells were involved in the typical movements and decisions made during ongoing behavior. As a result, the flood gates opened and our results began to be publishable; we suggested that the structure of a normal assembly among the striatal neurons during movement was disrupted in the dopamine depleted striatum. Furthermore, we showed that crude light activation of both cell types could mimic the turning and the disruption of striatal assemblies seen after the lesion of dopamine cells. In contrast, a more subtle pulsatile optical stimulation had the opposite effect and returned assembly dynamics to more normal levels [71].

The simple story that D1 cells say ”go” and D2 cells ”stop” is clearly very oversimplified. When we trained mice to reach for a chocolate pellet with one paw, interruption of the action of D1 cells, although it disturbed the early part of the reach, did not change much its initiation. In contrast, activation of D2 cells led animals to miss the target, but the movement stopped just fine [72]. This is in itself a simplification since, while the animals perform the task, connections between the two pathways can be modified, and in spite of the known anatomy, activity in both sides of the brain are important for the behavior.

## 3. Stepping away—A New Path

We have now begun to look beyond the cortical consequences of dopamine loss, particularly considering that animal models only address the end-stage of the disease. Bilateral lesions kill animals very quickly [73] unless they are given an equivalent of human patient intensive care [74]. Finding a cure is not going to be possible from this end-stage scene. My first attempts at a slower model of cell death really did not replicate the extent of the dopamine cell loss, even though the subthalamic overactivity, initiated by globus pallidus lesions in rats, caused a damage that rapidly progressed over weeks [75].

I had earlier resisted the genetic path of investigation because, although many genes are implicated in familial PD, only about 10% of patients have a clearly established inherited link. The newer Genome Wide Association Studies suggest that many genes associated with the disease might have been missed since some genes might have strong associations with the disease [76,77] and have high prevalence but low penetrance, making them hard to find in familial studies. In any event, accumulations of proteins, including alpha-synuclein, encased in lipid membranes, called Lewy bodies, were present in both the idiopathic majority, as well as in the familial minority. Although that could have made them a pathological identifier of PD, they also occur with a more extensive distribution in Lewy body dementia. My interest in protein metabolism changed, however, when I met a group who had developed a three-dimensional culture from human stem cells: an organoid. An additional benefit was immediately clear: their dopamine cells made neuromelanin [78] a great improvement from rodents! I saw that fact as a chance to move from studying how a whole brain responds to the loss of dopamine to closely examining how the cells die in the first place. If this method enables us to look at the ultimate causes of the disease, we should dedicate our effort where the best prospects are. In the rest of this review, I focus on the possible opportunities and problems associated to this newly accessible methodology.

## 4. More Things That Led My Way

In Okinawa Institute for Science and Technology (OIST), we had developed methods to build cortico-striatal cultures of mouse neurons [60]. These had solved some problems for us, but they were mouse neurons [79]. The organoids grew dopamine cells with neuromelanin; an early study indicating that neuromelanin coexisted with the extra vulnerability of dopamine cells [80] had intrigued me for years. Moreover, a recent paper describing genetically engineered neuromelanin in rat dopamine cells brought promise of a new model of the disease [81]. Having lived through the advent of antibodies to beta-amyloid for Alzheimer’s, I am not a fan of adding antibodies to alpha-synuclein as treatment for PD. It could be said that although the organoids have a genetic “disease” that only accounts for few of the patients, they could let us understand much more about alpha-synuclein and its role in dopamine cell death. Animal models have given confusing results: Chronic MPTP infusions in mice cause much less damage in alpha-synuclein knock-out mice [82]; conversely, expressing synuclein A53T, the mutation associated with PD, in otherwise normal mice, produces a severe dopamine depletion and alpha-synuclein accumulations [83]. It seems that most animal models do not develop typical Lewy bodies [84]. Perhaps only human cells, available in the organoids, can develop Lewy bodies with the typical morphology and biochemistry. Of course, one thing that organoids are not by far is whole brains; they have extracellular space and no blood–brain barrier and no microglia—unless they are added. They do have astrocytes and oligodendrocytes, and therefore, some experiments on their role might be possible. Perhaps glial cells in organoids also accumulate the leucine-rich repeat kinase 2 (LRRK2), as do astrocytes in patient brains [85]—just a sample of important things these strange cultures “organoids” provide to experimentalists, although above all, they allow access to human neurons and glia typical of midbrain, the very place where dopamine cells normally live.

For the future, the hope is that these model organoids will be more closely matched to the normal human brain and so will provide a better platform on which to test therapeutics for neurological diseases. It could be that the current failures in therapeutic developments are due to the tests having been done on rodent brains or in isolated cells, lacking the complexity of an interactive network, another characteristic of human brain cells. In principle, organoids grown from induced pluripotent human stem cells (iPSCs) expressing various genetic causes of the disease could provide the much-needed detailed pathology and, perhaps, a final common path. It is not at all a straightforward task: for example, it seemed that LRRK2 gain of function mutations were a cause of alpha-synuclein accumulation [86] and that inhibitors of the kinase activity reduced the accumulations [87]. There are even some kinase inhibitors that may be useful clinically [88], although knock-out animals with no LRRK2 also have alpha-synuclein accumulations [89]. In human genetic studies, it seems that loss-of-function mutations in LRRK2 are not associated with any disease; therefore, perhaps there is still hope in spite of the knock-out data [90]. In conclusion, clearly, there are many more interesting questions to answer in this area.

## 5. Was It a Wasted Life in Research, with Nothing to Show for It?

The fact that since 1969, PD patients’ life expectancy has shifted from 5 to 20 years is some justification for what we have been involved with. All of the pharmacology, anatomy, and neurophysiology that have been my life so far have contributed to treatment strategies, from L-DOPA itself (still the first line treatment), to surgical lesions, and deep brain stimulation, which have increased survival and reduced symptoms for patients. The idea that the brain compensated for injury was new in 1973, but now we are beginning to understand just how widespread it is, and how much of a mixed blessing it can be. Raisman’s experiments, which pioneered the idea that the brain did rewire after injury, had the synapses that were missing replaced by cells not normally making synapses at that site. Not so much a repair as a rewiring that did not have obvious behavioral consequences. Now we have ample evidence that the brain normally remodels itself, even during the formation of memories for instance [91,92]. As the molecular revolution proceeds, the genetic underpinning of repair in the nervous system has opened new opportunities. Recently, for example, new ways have been suggested that might allow us to speed up the recovery from stroke by creating an “excitable brain state” that encourages regrowth [93]. Perhaps a rescue of dopamine cells will require more subtlety because it needs to be more focused, but the expansion of the genetic causes of functional damage and recovery in the central nervous system now has a new focus and new tools with which to reach the target.

As we put 2020 behind us, 2021 might become a bright new year, not only for the responses to the COVID-19 pandemic, but for the generation of new targets for therapeutic interventions to save, or better to rescue, dopamine cells from their death, and patients, from the Parkinsonism that ruins their quality of life, until it finally ends it.
